# Immunity status of adults and children against poliomyelitis virus type 1 strains CHAT and Sabin (LSc-2ab) in Germany

**DOI:** 10.1186/1471-2334-10-347

**Published:** 2010-12-09

**Authors:** Maren Eggers, Elena Terletskaia-Ladwig, Holger F Rabenau, Hans W Doerr, Sabine Diedrich, Gisela Enders, Martin Enders

**Affiliations:** 1 Labor Prof. G. Enders & Partner and Institute of Virology, Infectious Diseases and Epidemiology e.V., Stuttgart, Germany; 2 Institute of Medical Virology, Hospital of the Johann Wolfgang Goethe University of Frankfurt, Frankfurt, Germany; 3 Robert Koch-Institute, Berlin, Germany

## Abstract

**Background:**

In October 2007, the working group CEN/TC 216 of the European Committee for standardisation suggested that the Sabin oral poliovirus vaccine type 1 strain (LSc-2ab) presently used for virucidal tests should be replaced by another attenuated vaccine poliovirus type 1 strain, CHAT. Both strains were historically used as oral vaccines, but the Sabin type 1 strain was acknowledged to be more attenuated. In Germany, vaccination against poliomyelitis was introduced in 1962 using the oral polio vaccine (OPV) containing Sabin strain LSc-2ab. The vaccination schedule was changed from OPV to an inactivated polio vaccine (IPV) containing wild polio virus type 1 strain Mahoney in 1998. In the present study, we assessed potential differences in neutralising antibody titres to Sabin and CHAT in persons with a history of either OPV, IPV, or OPV with IPV booster.

**Methods:**

Neutralisation poliovirus antibodies against CHAT and Sabin 1 were measured in sera of 41 adults vaccinated with OPV. Additionally, sera from 28 children less than 10 years of age and immunised with IPV only were analysed. The neutralisation assay against poliovirus was performed according to WHO guidelines.

**Results:**

The neutralisation activity against CHAT in adults with OPV vaccination history was significantly lower than against Sabin poliovirus type 1 strains (Wilcoxon signed-rank test P < 0.025). In eight sera, the antibody titres measured against CHAT were less than 8, although the titre against Sabin 1 varied between 8 and 64. Following IPV booster, anti-CHAT antibodies increased rapidly in sera of CHAT-negative adults with OPV history. Sera from children with IPV history neutralised CHAT and Sabin 1 strains equally.

**Conclusion:**

The lack of neutralising antibodies against the CHAT strain in persons vaccinated with OPV might be associated with an increased risk of reinfection with the CHAT polio virus type 1, and this implies a putative risk of transmission of the virus to polio-free communities. We strongly suggest that laboratory workers who were immunised with OPV receive a booster vaccination with IPV before handling CHAT in the laboratory.

## Background

To prevent and control the spread of nosocomial viral infections, disinfectants with proven virucidal efficacy must be used. Therefore, disinfectants must pass a virucidal activity test performed in compliance with good laboratory practise and country-specific standards. For instance, the European Committee for Standardization Technical Committee 216 - Chemical Disinfectants and Antiseptics (CEN/TC 216) developed a European standard that comprises a virucidal quantitative suspension test for chemical disinfectants and antiseptics used in human medicine (EN 14476:2005+A1:2006) [1]. According to the European standard EN 14476, Sabin poliovirus type 1 vaccine LSc-2ab is one of the test viruses. Generally, small non-enveloped viruses such as picornaviruses are very resistant to biocides. Therefore, poliovirus has been most widely used for virucidal testing. In October 2007, during a plenary meeting of the working group CEN/TC 216, it was suggested that the Sabin poliovirus type 1 vaccine LSc-2ab should be replaced by the poliovirus type 1 vaccine strain CHAT. The reason therefore were difficulties in the availability of the Sabin strain LSc-2ab in some European countries. In Germany, the virus can be obtained from the German Association for the Control of Viral Diseases [Deutsche Vereinigung zur Bekämpfung der Viruskrankheiten (DVV)]. Another source is the National Institute for Biological Standards and Control (NIBSC), offering the WHO reference strain Sabin Original Virus, second passage (SO+2) (NIBSC, code 01/528). In our laboratory, the original Sabin virus obtained from Behringwerke AG is used. In this study we used strains from all these three different sources to show that their properties are identical. Both attenuated vaccine strains for poliovirus type 1, CHAT and Sabin, were derived from the wild Mahoney strain. During the 1950 s, several virus variants were derived from the Mahoney strain by successive passages in various in vivo and in vitro cell substrates and selected for reduced neurovirulence in monkeys, to be used as vaccines [2-4]. Three live poliovirus type 1 vaccine strains have been generated. The best-known vaccine strain is probably the Sabin poliovirus type 1 strain (LSc-2ab), developed by Albert Bruce Sabin at the University of Cincinnati College of Medicine and Children's Hospital Research Foundation. The CHAT vaccine strain was produced by Hilary Koprowski at The Wistar Institute of Anatomy and Biology, Philadelphia. The CHAT virus and the third vaccine strain, Cox (also named the Lederle SM strain), are derived from a common progenitor SM N-90 strain and are genotypically more closely related to each other than to the Sabin virus LSc-2ab [4]. Both CHAT and Cox strains exhibit a higher degree of neurovirulence than Sabin 1 [4,5]. Because of the superior immunogenic and safety profile of Sabin strains, their use was widely recommended for oral polio vaccination (OPV) [5,6].

Jonas Salk, at the University of Pittsburgh, took a different approach and developed an inactivated poliovirus vaccine (IPV). The poliovirus strain used by Salk and still used by most manufacturers is poliovirus type 1 Mahoney. The Salk polio vaccine was introduced worldwide in 1955, but in the early 1960 s, IPV was replaced by OPV in many countries. In contrast to other European countries, in particular the Netherlands, Denmark, and the UK, IPV was not successfully implemented in Germany at this time and reached only 5 percent of its population in 1960. Wide vaccination against poliomyelitis was introduced in 1962 using the OPV containing Sabin strain LSc-2ab [7]. Germany changed from an OPV to an IPV vaccination schedule in 1998 because of the risk of vaccine-associated paralytic polio (VAPP). Between 1990 and 1999, 15 cases of VAPP had been registered in Germany [8]. IPV use, which carries no risk of VAPP, increased after 1999 also in other European countries and in the USA [9,10]. The majority of our laboratory staff was vaccinated before 1998 with Sabin strain LSc-2ab. In a previous study, the immunity status of 10 members of the laboratory staff with OPV history against CHAT was analysed using neutralisation test to estimate the potential risk of laboratory-associated infections and transmission to the community. Seven of the samples had the antibody titres against CHAT more than twofold lower than those against poliovirus type 1 Sabin, and three of them had the titres less than 8 [11]. In the following study we expanded the study group and compared immunity against CHAT and Sabin in adults with OPV history and in children vaccinated with IPV.

## Methods

### Serum samples

A total of 78 sera from 69 donors were used for this study.

#### Adults

Group I included sera from 41 adults. Sera from 29 adults were submitted to our laboratory in 2009 for determination of immunity status to poliovirus. Their vaccination history is not known, but we assumed, that they were vaccinated with OPV as the majority of German people born before 1998. In addition, sera from 12 laboratory workers potentially exposed to poliovirus were obtained between 2008 and 2009 by an occupational health physician according to the examination program of the German Workers Compensation Act (G 42 "Activities with a risk of infection"). The sera were stored at -20°C prior to testing. All laboratory workers provided written and/or oral informed consent prior testing. Ten laboratory workers were vaccinated with OPV only, from four to seven times according to the records in their immunization record cards. The vaccination history of three representative cases according to the immunization record card is given in Table 1. Schematic representation of the vaccination history of two other workers, who were vaccinated with both OPV and IPV is presented in Table 2. Nine sera from laboratory workers were sent to other laboratories to validate the results.

**Table 1:** Results of neutralization test with CHAT and Sabin 1 strains in paired sera of three laboratory workers with low antibody titers post OPV and four weeks later after IPV booster.

No.	Year of birth	Vaccination history	IPV booster 10/2009	Poliovirus Type 1 Strain CHAT ATTC VR-1562		Poliovirus Type 1 Strain Sabin (LSc-2ab) Behringwerke AG	
				GMT*	(IU/ml)	GMT	(IU/ml)
5a	1979	**OPV**:	before	< 8	(< 1,1)	32	(1,7)
5b		10/1979	after	512	(71,2)	3649	(281,1)
		01/1980					
		02/1981					
		02/1991					

6a	1959	**OPV:**	before	< 8	(< 1,1)	32	(1,7)
6b		vaccination5 xin Russia	after	287	(39,9)	1625	(125,2)

8a	1966	**OPV**:	before	< 8	(< 1,1)	16	(0,8)
8b		05/1966	after	575	(79,9)	575	(44,3)
		07/1966					
		08/1966					
		11/1972					
		01/1973					
		11/1978					
		01/1993					

GMT* Reciprocal geometric mean titers

**Table 2:** Immunization record of two laboratory workers vaccinated with OPV and IPV

**No**.	Year of birth	date of vaccination	Poliovirus Type 1 Strain CHAT	Poliovirus Type 1 Strain Sabin (LSc-2ab)
			ATTCVR-1562 GMT (IU/ml)	WHO-reference virus GMT (IU/ml)
28	1962	**IPV: **04/1964, 06/1964, 09/1964 **OPV: **10/1964, 12/64, 04/1964, 01/1971, 03/82	35 (4.9)	64 (3.4)

34	1985	Until 1991 OPV vaccination in Russia, **OPV: **04/1985, 05/1985, 07/1985, 01/1986, 03/1986, 02/1987, 03/1987, 11/1996 **IPV: **02/2002	256 (35.5)	512 (27.5)

#### Children

Group II consisted of 28 samples from children born after 1998, aged 1 to 10 years. Their sera were submitted to our laboratory in 2009 for determination of immunity status to poliovirus. Since 1998, only IPV is recommended to use in Germany. Therefore, we assumed, that these children had been vaccinated with IPV.

The study was carried out in compliance with the Helsinki Declaration. None of the samples was collected for study purposes. Discarded sera from our routine diagnostics were de-identified. Sera from laboratory workers were coded. All samples were tested in an anomysed fashion. Ethical approval was not required.

### Virus

The following poliovirus type 1 strains of passages 2-3 were used for challenge:

- Sabin original virus, second passage (SO+2) (Behringwerke AG, Marburg, Germany)

- WHO reference virus, Sabin original, second passage (NIBSC, code 01/528, Hertfordshire, UK)

- DVV reference virus, Sabin original, second passage (LSc-2ab) manufactured by Chiron-Behring, Marburg (Eurovir, Luckenwalde, Germany)

- CHAT strain (ATCC, catalogue no. VR-1562)

### Microneutralisation assay

Neutralising titres against poliovirus were detected according to WHO guidelines [12]. All serum samples were heat-inactivated (56°C/30 min). Sera and the positive and negative controls were serially two-fold diluted from 2 to 1024, and 25 μl of challenge virus was added to 25 μl of serum dilution, followed by incubation for 3 hours in a CO_2 _incubator. The challenge virus contained 100 TCID_50 _(range 50-200 TCID_50_/ml). After adding 0.1 ml of Hep2 cell suspension (European Collection of Cell Cultures, Salisbury, UK, used between passages 5 and 30), the plates were incubated in a CO_2 _incubator at 36°C for 5 days. After staining with crystal violet, plates were dried and read visually. Each test serum was investigated in duplicate on at least two separate occasions, and their titres did not vary more than two-fold between and within the assays. Reciprocal geometric mean titres (GMT) for each patient serum were calculated using all measured results. An in-house reference serum was included in each test run to convert the serum titres into international units (IU) and to control the reproducibility of results. Calibration of the in-house reference serum was carried out against the 2nd International Standard Poliovirus Antiserum (code 66/2020, NIBSC, Hertfordshire, UK) containing 25 IU for poliovirus type 1. For this purpose, the in-house reference serum and the 2nd International Standard Poliovirus Antiserum were titrated in parallel on six separate occasions.

### Statistics

Comparison of the data from two groups was accomplished by using Wilcoxon's signed-rank test and SYSTAT statistics computer software. Significance was defined as achieving a P value of 0.05.

## Results

### Calibration of in-house reference serum in international units

The GMT of the International Standard Antiserum and of the in-house reference serum calculated from all measured results obtained with strain Sabin from Behringwerke were 324 and 74, respectively. Accordingly, as the International Standard Antiserum contained 25 IU/ml, the potency of the in-house reference serum was 74/324 × 25 = 5.7 IU/ml. The geometric mean titre of the in-house reference serum was 55 in a neutralisation test against DVV Sabin 1 reference virus, 106 against WHO Sabin 1 reference virus, and 40 against CHAT. These titres were used to transform the results obtained with these strains into international units per millilitre.

A more than four-fold difference in the titre values achieved with different strains was considered significant. This was based, on the fact that the geometric mean titre of the in-house reference serum against WHO Sabin 1 reference virus was approximately two-fold higher than against the CHAT strain, and on the other hand, the fact that the acceptable range of neutralisation titres should be within one two-fold dilution, as recommended by the WHO [12].

### Poliovirus neutralising antibodies in adults

**Figure 1:**
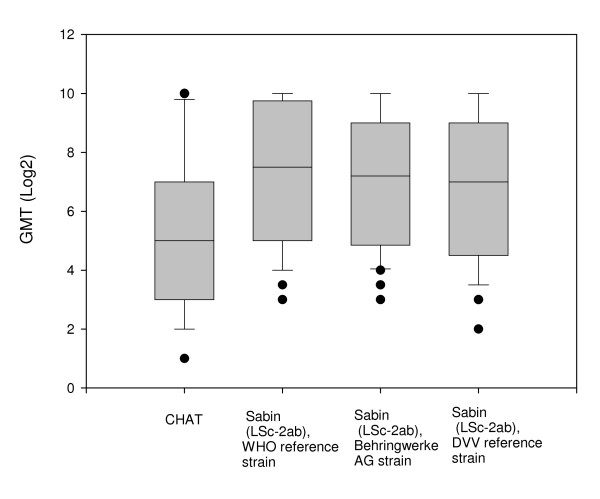
**Neutralizing antibodies against different strains of poliomyelitis virus type 1 in adults**. The boxplots show the 10th, 25th, 50th (median), 75th and 90th percentiles. The circles indicate outlier observations.

There was no significant difference between neutralising antibody titres found in the 41 sera from adults with three different stocks of the Sabin poliovirus type 1 strain when examined by the Wilcoxon signed-rank test (P > 0.25) (Figure 1). The antibody titre of each serum tested with different stocks of the Sabin poliovirus type 1 strain varied no more than three-fold. In contrast, the neutralisation activity against CHAT was significantly lower than against stocks of Sabin poliovirus type 1 (Wilcoxon signed-rank test P < 0.025). In 13 sera, the antibody titre against CHAT was fivefold and more lower than that against poliovirus type 1 Sabin. In eight sera, the titres against CHAT were even less than 8 (Table 3). However, in approximately half of the sera, the difference between anti-CHAT and anti-Sabin 1 antibody concentration was not more than four-fold.

**Table 3:** Results of neutralization test with different poliovirus type 1 challenge strains in adults immunized totally or partially with OPV.

			Poliovirus Type 1 Strain CHAT	Poliovirus Type 1 Strain Sabin (LSc-2ab)		
				
			ATTC VR-1562	WHO reference virus	Behringwerke AG	DVV reference virus
		
Case No.	Age	Ratio*	GMT* (IU/ml)	GMT (IU/ml)	GMT (IU/ml)	GMT (IU/ml)
1	16		< 8 (< 1,1)	11 (0,6)	11 (0,8)	8 (0,8)
2	29		< 8 (< 1,1)	16 (0,8)	19 (1,5)	11 (1,1)
3	24		< 8 (< 1,1)	16 (0,8)	19 (1,5)	8 (0,8)
4	33		< 8 (< 1,1)	8 (0,4)	8 (0,6)	< 8 (< 1,1)
5	29		< 8 (< 1,1)	32 (1,7)	53 (4,1)	32 (3,3)
6	49		< 8 (< 1,1)	32 (1,7)	19 (1,5)	16 (1,6)
7	42		< 8 (< 1,1)	45 (2,4)	45 (3,5)	64 (6,6)
8	43		< 8 (< 1,1)	16 (0,8)	11 (0,8)	11 (1,1)
						
9	50	8	8 (1,1)	64 (3,4)	16 (1,2)	32 (3,3)
10	53	11	8 (1,1)	90 (4,8)	53 (4,1)	32 (3,3)
11	10	11	16 (2,2)	181 (9,7)	256 (19,7)	181 (18,7)
12	37	10	17 (2,4)	181 (9,7)	76 (5,9)	128 (13,2)
13	40	17	28 (3,9)	512 (27,5)	362 (27,9)	256 (26,5)
14	31	8	32 (4,4)	256 (13,7)	152 (11,7)	256 (26,5)
15	40	32	32 (4,4)	1024 (55)	362 (27,9)	362 (37,5)
16	44	5	45 (6,2)	256 (13,7)	181 (13,9)	181 (18,7)
17	40	11	64 (8,8)	724 (38,9)	430 (33,1)	724 (75)
18	28	5	128 (17,7)	724 (38,9)	304 (23,4)	256 (26,5)
19	18	5	128 (17,7)	724 (38,9)	512 (39,4)	512 (53)
20	37	8	128 (17,7)	1024 (55)	861 (66,3)	512 (53)
21	49	8	128 (17,7)	1024 (55)	1024 (78,9	1024 (106,1)
						
22	41	4	8 (1,1)	32 (1,7)	32 (2,5)	22 (2,3)
23	43	4	8 (1,1)	32 (1,7)	26 (2,0)	22 (2,3)
24	25	1	16 (2,2)	16 (0,8)	22 (1,7)	16 (1,6)
25	59	4	16 (2,2)	64 (3,4)	38 (2,9)	32 (3,3)
26	54	1	32 (4,4)	32 (1,7)	26 (2,0)	16 (1,6)
27	64	2	32 (4,4)	64 (3,4)	53 (4,1)	22 (2,3)
28	46	1	35 (4,9)	64 (3,4)	64 (4,9)	64 (6,6)
29	60	1	64 (8,8)	90 (4,8)	76 (5,9)	90 (9,3)
30	29	2	64 (8,8)	181 (9,7)	181 (13,9)	64 (6,6)
31	60	2	128 (17,7)	256 (13,7)	215 (16,6)	181 (18,7)
32	46	2	128 (17,7)	256 (13,7)	152 (11,7)	128 (13,2)
33	36	2	128 (17,7)	362 (19,4)	256 (19,7)	256 (6,5)
34	24	2	256 (35,5)	512 (27,5)	512 (39,4)	512 (53)
35	28	4	256 (35,5)	1024 (55)	724 (55,8)	512 (53)
36	37	2	512 (71,1)	1024 (55)	1024 (78,9)	1024 (106,1)
37	58	2	512 (71,1)	1024 (55)	1024 (78,9)	724 (75)
38	35	1	1024 (142,3)	1024 (55)	724 (55,8)	1024 (106,1)
39	36	1	1024 (142,3)	1024 (55)	861 (66,3)	724 (75)
40	40	1	1024 (142,3)	1024 (55)	1024 (78,9)	1024 (106,1)
41	61	1	1024 (142,3)	1024 (55)	1024 (78,9)	1024 (106,1)

The sera were grouped as follows:

In sera No. 1 to 8 antibody titres against the strain CHAT were lower than 1: 8.

In sera No. 9 to 21 antibodies titre against the strain CHAT were five or more fold lower than against the strain Sabin.

In sera from No. 22 to 41 the difference between antibodies titre against the strain CHAT and the strain Sabin was fourfold and less.

Case numbers in Tables 1 and 2 correspond to the case numbers in Table 3.

*Ratio of titre against Sabin 1 WHO reference strain to titre against CHAT

**GMT Reciprocal geometric mean titers

Only an one or two-fold difference between anti-CHAT and anti-Sabin 1 titres was found in the sera of two laboratory workers who had a history of mixed OPV and IPV vaccination (Table 2).

Three laboratory workers vaccinated with OPV, from four to seven times according to the records in their immunization record cards, had antibody titres against CHAT under 8. Their antibody titres increased significantly after booster vaccination with IPV (Table 1).

Sera from nine laboratory workers were sent to the national reference laboratory for polio- and enteroviruses at the Robert Koch Institute (RKI) in Berlin and to the Institute for Medical Virology, Johann Wolfgang Goethe University, Frankfurt, to verify our results. The obtained data are presented in Table 4. Case numbers in Table 4 correspond to the case numbers in Table 3. Some sera had been used up in the first experiments, so the paired sera were collected. In Table 1 and 4, these sera are marked with the letter b. All laboratories measured lower anti-CHAT than anti-Sabin 1 titres in both their internal reference sera and patient sera. To recalculate the RKI results from titres to international units, the GMT of the RKI internal in-house reference serum tested with Sabin 1 (140) and CHAT (48) on six separate occasions according to WHO recommendations was used [12]. The RKI internal in-house reference serum had been calibrated earlier against the 2nd International Standard Poliovirus Antiserum with a result of 3.0 IU/ml [13]. Transformation of the titre values obtained in the Institute for Medical Virology of Frankfurt to international units was performed using the internal in-house reference serum of the laboratory of Prof. Enders. This in-house reference calibrated earlier against the International Standard Antiserum as described above was included in every assay run in Frankfurt. The GMT of the serum calculated from all measured results was 22 for CHAT and 86 for Sabin 1.

**Table 4:** Results of neutralization test with poliovirus type 1 strain CHAT and Poliovirus Type 1 Strain Sabin (LSc-2ab) in three different laboratories.

	Laboratory Nr. 1			Laboratory Nr. 2			Laboratory Nr. 3		
			
Case **No**.	CHAT ATTC VR-1562	Sabin 1 WHO reference strain	Ratio*	CHAT ATTC VR-1562	Sabin 1 WHO reference strain	Ratio	CHAT ATTC VR-1562	Sabin 1 WHO reference strain	Ratio
	GMT** (IU/ml)	GMT (IU/ml)		GMT IU/ml	GMT IU/ml		GMT IU/ml	GMT IU/ml	
7b	< 8 (< 1,1)	91 (4,9)	> 4,5	12 (0,6)	192 (4,1)	7,1	< 10 (< 1,3)	80 (5,3)	> 4,1
9	8 (1,1)	64 (3,4)	3,1	32 (1,5)	128 (2,7)	1,8	< 10 (< 1,3)	40 (2,7)	> 2,0
12b	23 (3,2)	362 (19,5)	6,1	64 (3,1)	256 (5,5)	1,8	10 (2,6)	160 (10,6)	4,1
13b	32 (4,4)	> 786 (> 42,3)	> 9,4	48 (2,3)	> 512 (> 11)	> 4,7	20 (5,2)	640 (42,4)	8,2
28b	32 (4,4)	64 (3,4)	0,8	96 (4,6)	128 (2,7)	0,6	20 (5,2)	40 (2,7)	0,5
16b	64 (8,9)	512 (27,5)	3,1	128 (6,2)	512 (11,0)	1,8	20 (5,2)	320 (21,2)	4,1
21	128 17,8	2048 110,1	6,2	384 (18,6)	> 512 (> 11)	> 0,6	40 (10,4)	1280 (84,8)	8,2
8b	256 (35,6)	362 (19,5)	0,5	512 (24,8)	512 (11,0)	0,4	80 (20,7)	320 (21,2)	1,0
34b	256 (35,6)	> 786 (> 42,3)	> 1,9	> 512 (> 25)	> 512 (> 11)	-	80 (20,7)	320 (21,2)	1,0

**Internal control**	41 (5,7)	106 (5,7)	1,0	62 (3)	140 (3)	1,0	22 (5,7)	86 (5,7)	1,0

Laboratory Nr. 1 - Labor Enders, Stuttgart, Laboratory Nr. 2 - Robert Koch-Institute (RKI), Berlin, Laboratory Nr. 3 - Institute for Medical Virology, Johann Wolfgang Goethe-University of Frankfurt

*Ratio of IU/ml Sabin to IU/ml CHAT

**GMT Reciprocal geometric mean titers

A greater than four-fold difference was observed in international unit values between anti-CHAT and anti-Sabin 1 neutralisation antibodies in sera nos. 7b, 13b, and 21.

### Poliovirus neutralising antibodies after history of IPV in children

**Figure 2:**
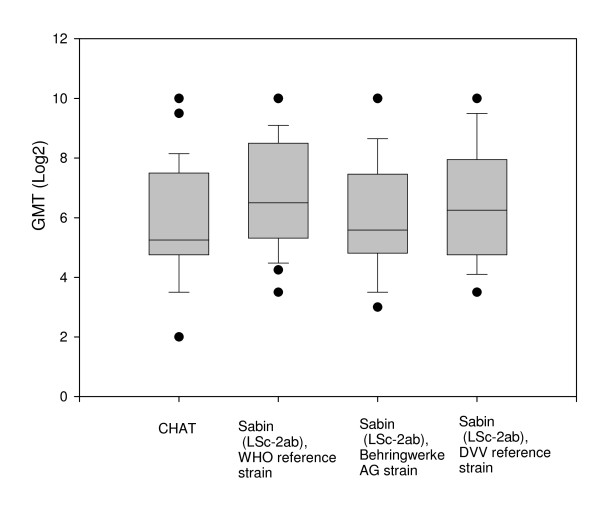
**Neutralizing antibodies against different strains of poliomyelitis virus type 1 in children**. The boxplots show the 10th, 25th, 50th (median), 75th and 90th percentiles. The circles indicate outlier observations.

The 28 sera from children equally neutralised CHAT und Sabin strains (Wilcoxon signed-rank test P > 0.1) (Table 5; Figure 2).

**Table 5:** Results of neutralization test with different poliovirus type 1 challenge strains in 28 children immunized with IPV.

			Poliovirus Type 1 Strain CHAT		Poliovirus Type 1 Strain Sabin (LSc-2ab)					
				
			ATTC VR-1562		WHO-reference virus		DVV-reference virus		Behringwerke AG	
Case No.	Age	Ratio*	GMT**	IU/ml	GMT	IU/ml	GMT	IU/ml	GMT	IU/ml
42	8		< 8	< 8	23	1,3	11	0,9	16	1,7
43	10	1	11	1,7	11	0,6	11	0,9	8	0,9
44	9	2	11	1,7	27	1,5	18	1,5	11	1,2
45	6	1	16	2,4	19	1,1	20	1,6	11	1,2
46	5	1	16	2,4	23	1,3	23	1,8	16	1,7
47	9	2	19	2,9	38	2,2	25	2,1	27	2,9
48	3	2	25	3,8	51	2,9	37	3,0	32	3,5
49	5	1	32	4,8	32	1,8	23	1,8	23	2,5
50	9	1	32	4,8	45	2,6	32	2,6	32	3,5
51	8	2	32	4,8	64	3,6	64	5,2	45	4,9
52	8	2	32	4,8	64	3,6	64	5,2	45	4,9
53	10	3	32	4,8	91	5,1	64	5,2	64	7,0
54	5	3	32	4,8	81	4,6	97	7,9	51	5,5
55	8	4	32	4,8	128	7,2	128	10,4	91	9,9
56	4	3	45	6,8	128	7,2	128	10,4	64	7,0
57	8	2	64	9,6	128	7,2	64	5,2	32	3,5
58	10	1	64	9,6	64	3,6	91	7,3	45	4,9
59	2	1	91	13,6	91	5,1	45	3,7	45	4,9
60	3	1	128	19,2	181	10,2	181	14,7	91	9,9
61	8	1	181	27,2	181	10,2	128	10,4	64	7,0
62	3	1	181	27,2	256	14,5	256	20,8	181	19,7
63	3	3	181	27,2	512	29,0	256	20,8	181	19,7
64	9	3	181	27,2	512	29,0	512	41,5	256	27,9
65	8	3	181	27,2	512	29,0	724	58,7	362	39,5
66	1	2	203	30,5	406	23,0	223	18,1	161	17,6
67	4	2	256	38,4	512	29,0	256	20,8	181	19,7
68	4	1	724	108,6	1024	58,0	1024	83,0	1024	111,7
69	1	1	1024	153,6	1024	58,0	724	58,7	1024	111,7

*Ratio of titre against Sabin 1 WHO reference strain to titre against CHAT

**GMT Reciprocal geometric mean titers

## Discussion

The neutralising antibody test is the method of choice to determine the immune status against poliovirus. Although the precise concentration of antibodies that is necessary for protection is unknown, it is accepted that any level of type-specific neutralisation of infection in cell culture is protective [14]. In the WHO Manual for the virological investigation of polio, titration of sera from 8 to 1024 is recommended [1]. Therefore, in practise, this is a titre of 8. According to this study, all adults were protected against the poliovirus type 1 Sabin strains. In contrast, one-fifth of the tested adult persons had an antibody titre against CHAT under 8 and presumably were not protected. Obviously, the difference between neutralising titres against type 1 Sabin strain and CHAT base on the antigenic properties of strains and not on the variations in test performance such as viral dose and fluctuations in the titre of antibodies. This claim is supported by the fact that the adults were protected against all three type 1 Sabin strains from different sources but not against CHAT.

In addition, one-fourth of the CHAT positive sera had an antibody titre against CHAT four-fold and more lower than that against Sabin (nos. 8-18). Other laboratories also confirmed lower titre for CHAT in several samples. Moreover, the titre values measured by different laboratories were transformed into international units because the use of relative potency reduces the variation between laboratories, in contrast to direct titre estimation [15]. However, the relative potencies to Sabin 1 of two samples (7b and 13b) (Table 4) were still more than four-fold higher than to CHAT.

The fact, that the OPV Sabin strain-induced antibodies neutralise better the Sabin strain than the CHAT strain can be explained by the antigenic differences in these strains. The immunogenicity of poliovirus is determined by four distinct sites. The analysis of antigenic structures of CHAT and Mahoney with a panel of Sabin 1 neutralising monoclonal antibodies shows, that neither Mahoney or CHAT strains were neutralised by three antibodies against sites 1 and 3. CHAT alone did not react with two antibodies against site 2a. The results were consistent with known amino acid differences in these sites [16]. A clear difference between reactivity of inactivated Mahoney and live Sabin 1 was also demonstrated by monoclonal antibody-based block-ELISA [17].

Our findings raise the question of whether persons with low levels of neutralising antibodies to Sabin 1 are protected against reinfection with the CHAT strain. In the early years of polio vaccine development, many studies were carried out to determine the protective levels of polio antibodies [18]. Different studies indicated that persons with low serum neutralising antibody titres can be reinfected with wild as well with vaccine virus [19-22]. On the other hand, these studies suggested that persons with low but detectable antibodies are probably not in danger of developing clinical poliomyelitis. The lifelong protection to poliomyelitis when the titre falls below detectability can be explained with immunological memory. The secondary response to infection seems to be rapid enough to protect against paralytic disease. However, a more recent study carried out in 2005 in the Netherlands demonstrated that poliovirus-specific memory immunity in seronegative elderly people does not protect against virus excretion [23]. Abbink et al. challenged 429 elderly people with monovalent oral poliovirus vaccine (type 1 or 3) and followed immune response and virus excretions for 8 weeks. Most seronegative participants (81%) excreted poliovirus type 1 for 3 to 49 days. A neutralising effect of preexisting antibodies could be clearly seen: only 17% of prevaccinated and 22% of the naturally immunised persons excreted poliovirus type 1. Moreover, the period of excretion was shorter in persons with preexisting immunity (maximum 28 days). However, this indicates that immunised laboratory workers with low antibody titres can be reinfected with CHAT and may be a source of infection to others who have not been vaccinated. Because CHAT is characterised by a lower degree of attenuation and temperature-sensitivity than Sabin 1 and also shows molecular and biological properties closer to wild poliovirus strain, it has been strongly recommended that laboratories always use Sabin 1 from material that complies with WHO requirements for the production of oral polio vaccine [Martin J personal communication]. Such preparations are available from the National Institute for Biological Standards and Control (NIBSC), a centre of the Health Protection Agency (HPA) in the United Kingdom (ref. N0. 01/528, contact: Javier.Martin@nibsc.hpa.org.uk).

In our study, anti-CHAT antibodies increased rapidly after IPV booster in sera of three of the workers with OPV history. Moreover, children immunised with IPV had the same level of antibodies against CHAT as against Sabin 1. This result supplies an additional argument for the benefit of IPV using Mahoney strain, considering that CHAT is more similar to wild virus than Sabin 1, the one used for OPV. However, this conclusion has some limitations, since the study does not include similar subject groups: OPV in adults, versus IPV for children. Some adults were probably vaccinated a long time ago. There are different arguments for and against using of OPV or IPV. According WHO recommendations, during the pre-eradication period the national choice of vaccines must include OPV or IPV, or a combination of both [24].

## Conclusion

In any case, laboratory workers should be boostered with IPV before working with CHAT if it is not possible to avoid the use of CHAT for the research aims. The use of the CHAT strain would not comply to current requirements for the containment of poliovirus recommended by WHO [25]. CHAT should not be used for any standard laboratory assays including determination of virucidal efficiency. At the same time, it should be considered to replace poliovirus with an other virus (e.g. Echovirus 1 or an animal parvovirus), to test the virucidal efficacy of disinfectants. The use of poliovirus will be more restricted after eradication. Therefore, the validation of virucidal tests using another virus strain should be started as soon as possible.

## Competing interests

The authors declare that they have no competing interests.

## Authors' contributions

All authors contributed to the conception and analysis of data. All authors are involved in drafting the manuscript.

## Pre-publication history

The pre-publication history for this paper can be accessed here:

http://www.biomedcentral.com/1471-2334/10/347/prepub
